# Effects of Dietary Supplementation with 2-Hydroxy-4-(methylthio)-butanoic Acid Isopropyl Ester as a Methionine Supplement on Nitrogen Utilization in Steers

**DOI:** 10.3390/ani11113311

**Published:** 2021-11-19

**Authors:** Yuchao Zhao, Md Sazzadur Rahman, Mengmeng Li, Guangyong Zhao

**Affiliations:** State Key Laboratory of Animal Nutrition, College of Animal Science and Technology, China Agricultural University, Beijing 100193, China; yuchao@cau.edu.cn (Y.Z.); sazzad@cau.edu.cn (M.S.R.); limeng2021@cau.edu.cn (M.L.)

**Keywords:** cattle, methionine hydroxy analog, nitrogen retention, plasma metabolome profiling

## Abstract

**Simple Summary:**

Ruminants excrete large amounts of nitrogen (N), which negatively affects the environment. Supplying limiting amino acid (AA) methionine (Met) usually considerably improves the N utilization rate of ruminants. 2-Hydroxy-4-(methylthio)-butanoic acid isopropyl ester (HMBi) has been widely used as a Met supplement to ruminants. However, the effects of HMBi on the N utilization rate in ruminants usually vary widely. The experiment assessed the effects of dietary addition with HMBi on the N metabolism in beef steers and elucidated the mechanism using the plasma metabolome. Adding HMBi upregulated the plasma concentration of Met but did not improve the N utilization rate in steers. The main reason for the results could be that steers used in the experiment were in the fattening period. It is suggested to evaluate the effects of dietary addition with HMBi using growing cattle.

**Abstract:**

The objective of the experiment was to investigate the effects of dietary supplementation with 2-hydroxy-4-(methylthio)-butanoic acid isopropyl ester (HMBi) on the nitrogen (N) metabolism in beef steers. The plasma metabolites analyzed by metabolome profiling were used to clarify the impact mechanism. Three Simmental steers (body weight, 593 ± 23 kg) were used as experimental animals. Three levels of HMBi (i.e., 0, 12, and 24 g d^−1^) were added in a basal ration as experimental treatments. The steers and the dietary treatments were randomly allocated in a 3 × 3 Latin square design. The results showed that supplementing HMBi up to 24 g d^−1^ did not affect the N retention and N retention rate (NRR), and the fecal N/urinary N ratio even though it tended to linearly increase the uric acid N/urinary N ratio in steers. The results of plasma metabolome profiling showed that supplementing HMBi at 24 g d^−1^ upregulated the plasma concentrations of L-methionine (Met); Met-related metabolites including betaine, Met sulfoxide, and taurine; and L-isoleucine and tyrosine, whereas it downregulated L-serine, glycine, diaminopimelic acid, and other metabolites. The reason for the nonsignificant effect of HMBi on improving the N utilization in steers could be that the steers used in the experiment were in the fattening period. It is suggested to evaluate the effects of the dietary addition of HMBi using growing cattle in further research.

## 1. Introduction

The nitrogen retention rate (NRR) of beef cattle is the lowest among main domestic animals [1]. About 75–80% of the nitrogen (N) ingested by beef cattle is excreted in feces and urine [2]. A large amount of N excreted to the environment from beef cattle not only represents economic losses but also negatively affects the environment due to emissions of ammonia (NH_3_) and nitrous oxide (N_2_O) [3]. Hence, increasing NRR and decreasing N excretion are important objectives in beef cattle nutrition.

Methionine (Met) and lysine (Lys) are the first two limiting amino acids (AA) for ruminants fed with corn-silage-based diets [4]. Previous studies showed that balancing the dietary AA profile by supplementing Met to the diets of beef cattle improved N retention and NRR [5]. Since free Met was extensively degraded in rumen, dietary supplementation with free Met had a minor effect on improving the NRR in beef cattle [6]. To avoid the microbial degradation of free Met in the rumen, coated Met with polymer (2-vinylpyridine-co-styrene) and stearic acid [7] or ethyl cellulose and stearic acid [8] is usually supplemented to cattle. Other forms of rumen-protected Met are Met hydroxy analogs, including 2-hydroxy-4-(methylthio)-butanoic acid (HMB) and isopropyl ester of HMB (HMBi) [6,9]. HMBi can be absorbed across the rumen wall into the blood, converted to Met in the liver, and further utilized for protein synthesis in ruminants [10]. Some studies indicated that supplementing HMBi to the diets of dairy cows at 0.35% dry matter (DM) [11] and 0.18% DM [12] increased the milk yield and milk protein concentration. Supplementing HMBi to the diets of dairy cows at 0.13% DM reduced the N excretion and improved the N utilization rate [13]. Supplementing HMBi to the diet of growing Holstein steers at 15 or 25 g d^−1^ improved the daily liveweight gain and the plasma total protein concentration [14]. Other studies, however, showed that supplementing HMBi to the diets of dairy cows at 0.26% DM had no effect on the milk protein yield [15], and supplementing HMBi at 0.17% DM [16] and 0.20% DM [17] did not affect the N excretions. The inconsistent effects of HMBi on the N utilization in cattle among different experiments could be attributed to two aspects. One could be the extent of dietary Met supply to meet the Met requirement. Another could be that the utilization rate of HMBi varies among cattle in different growing stages. The objectives of the experiment were to evaluate the effects of dietary supplementation with HMBi on the N retention and NRR in steers and clarify the impact mechanism of HMBi using plasma metabolome profiling.

## 2. Materials and Methods

### 2.1. Animals, Treatments, and Experimental Design

The experimental procedures were approved by the Animal Care and Use Committee of China Agricultural University (20160611-2).

Three 29-month-old Simmental steers with an average body weight (BW) of 593 ± 23 kg were used as experimental animals. The steers were fed with a basal diet (Table 1) formulated according to the Nutrient Requirements and Feeding Standards of Beef Cattle [18]. The diet contained 12.53% CP and 6.40 MJ kg^−1^ net energy for maintenance and fattening (NEmf) (DM basis), which supplied about 1.20 and 1.30 times of maintenance requirements for CP and NEmf, respectively, with an expected daily liveweight gain of 0.40 kg d^−1^.

Each steer was supplied with 6.99 kg DM of the diet daily (including 14 kg corn silage and 4 kg concentrate mixture), which was about 90% of the ad libitum feed intake. No feed residuals of the steers were left in the whole experiment. Three levels of HMBi (minimum guarantee of 57% HMBi monomers) (i.e., 0, 12, and 24 g d^−1^) were added in the basal diet, respectively, as treatments. The steers and treatments were allocated in a 3 × 3 Latin square design. Each experimental period included 14 days for adaptation and 5 days for sampling.

The steers were housed in a tie-stall barn with rubber mats. The basal diet was prepared as a total mixed ration (TMR) immediately prior to feeding. Fresh drinking water was freely available. The BW of each steer was recorded at the beginning and the end of each experimental period in the morning before feeding and drinking water.

### 2.2. Sampling

Total urine and feces from each steer were collected and recorded daily. The feces excreted on the mat from each steer were collected immediately into a plastic bucket with a cover. An amount of 2% of the total feces was sampled and mixed with 20 mL sulfuric acid (2 *M*). The urine from each steer was collected using a rubber funnel fitted to the animal and connected to a plastic bucket added with 500 mL sulfuric acid (2 *M*) and surrounded with ice packs. An aliquot of 1% of the total urine was taken as the sample. The TMR was also sampled daily. On the last day of each experimental period, blood was taken from the jugular vein of each steer using a vacutainer tube containing K_2_EDTA (Greiner Bio-One, Frickenhausen, Germany) 3 h after feeding in the morning. Plasma samples were obtained after blood samples were centrifuged at 2000× *g* for 15 min at 4 °C. All samples were stored at −20 °C.

### 2.3. Determinations and Chemical Analyses

All TMR and fecal samples were lyophilized at −80 °C for 72 h using a freeze dryer (LGJ-12; Beijing Songyuan Huaxing Technology Development Co., Ltd., Beijing, China). The TMR was ground through a sieve with a pore size of 1 mm (FW177, Tianjin Taisite Instrument Co., Ltd., Tianjin, China). The fecal samples were ground using a mortar and a pestle.

The DM of feeds and feces was determined according to AOAC [21] using method no. 930.15. The crude ash and the CP (N × 6.25) were analyzed according to AOAC [21] using method nos. 942.05 and 984.13, respectively. The organic matter (OM) was calculated as the difference between DM and crude ash. The neutral detergent fiber (aNDF) and the acid detergent fiber (ADF) were analyzed using the methods of Van Soest et al. [22] on an ANKOM A200 semiautomatic analyzer (Ankom Technology Corp., Macedon, NY, USA). Heat-stable α-amylase and sodium sulfite were used for NDF analysis. The gross energy (GE) of TMR was analyzed using an oxygen bomb calorimeter (Parr 6300 Calorimeter; Parr Instrument Company, Moline, IL, USA). The AA of the basal diet were analyzed using a Hitachi L-8900 AA analyzer (Hitachi Co. Ltd., Tokyo, Japan). The rumen degradable protein (RDP) and the rumen undegradable protein (RUP) were calculated according to NRC [19] based on the CP fractionation (nonprotein N, soluble CP, neutral detergent insoluble CP, acid detergent insoluble CP) of feeds determined using the methods of Licitra et al. [20].

The N in urine was analyzed using the Kjeldahl method according to AOAC [21] using method no. 984.13. The urea and creatinine in urine were analyzed using commercial kits (Beijing Solarbio Science & Technology Co., Ltd., Beijing, China). The allantoin and uric acid in urine were analyzed according to the methods of Chen and Gomes [23]. The hippuric acid in urine was analyzed according to China Hygienic Standard (WS/T52-1996) [24]. The urea, creatinine, allantoin, uric acid, and hippuric acid in urine were analyzed on a UV–visible spectrophotometer (UV-1801; Beijing Rayleigh Analytical Instruments Co. Ltd., Beijing, China).

The plasma concentrations of total protein, albumin, glucose, total cholesterol, triglyceride, urea, uric acid, and creatinine were analyzed using commercial kits (Biosino Bio-Technology and Science Incorporation, Beijing, China) on an automatic biochemical analyzer (BS-420; Shenzhen Mindray Biomedical Electronic Co., Ltd., Shenzhen, China).

### 2.4. Metabolomics Analysis

The plasma samples for metabolomics analysis were prepared according to the description of Liu et al. [25]. Briefly, a volume of 100 μL of each thawed plasma sample was transferred into a centrifuge tube and added with 300 μL methanol and 10 μL internal standard (DL-o-chlorophenylalanine). Each sample was vortexed for 30 s and then centrifuged at 13,800× *g* for 10 min at 4 °C. The supernatant was transferred to a vial for LC–MS analysis.

Plasma metabolites were analyzed on an ultra-high-pressure liquid chromatography (UPLC) system (Thermo Fisher Scientific, Waltham, MA, USA) equipped with a column of BEH C18 (100 × 2 mm, 1.7 μm particle size, Waters, Milford, CT, USA) connected to a mass spectrometer (MS; TripleTOF 6600, AB Sciex, Framingham, MA, USA). The SIEVE software (Thermo Fisher Scientific, Waltham, MA, USA) was used to analyze the data of MS/MS spectra. The Kyoto Encyclopedia of Genes and Genomes Database (http://www.genome.jp/kegg, accessed on 30 November 2020) and the online Human Metabolome Database (http://www.hmdb.ca, accessed on 30 November 2020) were used to align the data to identify metabolites and the retention time index.

### 2.5. Calculations

The rumen microbial N flow to the steers was predicted according to Chen and Gomes [23] based on the total urinary excretions of purine derivatives (PD) including uric acid and allantoin:

Y = 0.85X + 0.385BW^0.75^ (1)

where Y refers to the total urinary purine derivatives (PD), mmol d^−1^; X, the absorbed PD, mmol d^−1^; BW^0.75^, the metabolic liveweight of steers, kg; 0.85, the recovery rate of absorbed purines as PD in urine; and 0.385, the endogenous excretion of PD, mmol kg^−1^ BW^0.75^ d^−1^.

Microbial N = (X × 70)/(0.116 × 0.83 × 1000) = 0.727X(2)

where the unit for microbial N is g d^−1^; X refers to the absorbed PD, mmol d^−1^; 70, the N content of purines, mg N mmol^−1^; 0.116, the purine N/total N ratio in mixed rumen microbes; 0.83, the digestibility of microbial purines.

### 2.6. Statistical Analysis

The data of N metabolism and conventional plasma indices were analyzed using PROC MIXED of SAS 9.4 (SAS Institute Inc., Cary, NC, USA) using the model:

Y_ijk_ = μ + T_i_+ P_j_+ S_k_ + e_ijk_ (3)

where Y_ijk_ refers to the dependent variable; μ, the overall mean; T_i_, the fixed effect of the *i*th treatment (i = 1 to 3); P_j_, the random effect of the *j*th period (j = 1 to 3); S_k_, the random effect of the *l*th steer (k = 1 to 3); and e_ijk_, error residue.

Orthogonal contrasts were applied to determine the linear and quadratic effects of HMBi levels. The least square means were compared using Tukey’s test for multiple comparisons. The treatment effects were declared to be significant at *p* ≤ 0.05 and a tendency at 0.05 < *p* ≤ 0.10. The metabolomics data were analyzed using SIMCA-P 14.0 (Umetrics, Umea, Sweden) for the orthogonal partial least squares discriminant analysis (OPLS-DA). Differentially expressed metabolites (DEMs) were used to perform the pathway enrichment using MetaboAnalyst 4.0 (http://www.metaboanalyst.ca, accessed on 30 November 2020) [26]. The DEMs between two groups were identified according to variable importance in projection (VIP) > 1 and false discovery rate (FDR) < 0.05.

## 3. Results and Discussion

Met is one of the first two limiting AA for protein synthesis in beef cattle [4]. As a hydroxylated analog of Met, about half of HMBi ingested by cattle was degraded by rumen microorganisms [27], and half was absorbed into the blood through the rumen wall and subsequently converted into Met in the liver [28,29].

Table 2 shows that the Met content of the basal diet (control group) was lower than the Met requirement of the steers. Supplementing 12 or 24 g HMBi d^−1^ to the basal diet increased the total Met content of the diet, which exceeded the Met requirement of the steers. However, the results in Table 3 show that supplementing 12 or 24 g HMBi d^−1^ (0.17% and 0.34% DM) did not affect the total N excretion (*p* > 0.05), N retention and NRR (*p* > 0.05), and the urinary excretions of nitrogenous components, including urea, uric acid, allantoin, creatinine, and hippuric acid (*p* > 0.05), even though it tended to linearly increase the uric acid N/urinary N ratio (*p* = 0.069) (Table 4). The results in the present experiment were in agreement with those of Chen et al. [16], who reported that supplementing HMBi at 0.17% DM did not affect the N utilization rate in lactating cows fed a diet (15.6% CP, DM basis) mainly composed of high-moisture corn, alfalfa, and corn silages. Whelan et al. [17] reported that dietary addition with HMBi at 0.20% DM did not affect the N excretions in feces, urine, and milk in dairy cows fed a diet based on corn silage or grass silage.

The results of the present experiment also showed that supplementing HMBi at 12 or 24 g d^−1^ did not affect the plasma concentrations of total protein, albumin, glucose, total cholesterol, triglyceride, urea, uric acid, and creatinine (Table 5). The results were in agreement with Čermáková et al. [31] who reported that supplementing HMBi at 0.16% DM did not affect the plasma concentrations of total protein and urea in dairy cows fed a diet based on alfalfa, corn silages, and ensiled corn cobs. However, Han et al. [14] reported that supplementing HMBi at 25 g d^−1^ increased the serum concentrations of total protein and albumin and decreased the serum concentration of urea in steers fed a diet based on corn silage and corn. The discrepancy in the results could be resulted from the extent of dietary Met supply (including Met from HMBi) to meet the requirements of animals among different experiments.

Most part of the HMBi that is not absorbed is hydrolyzed to 2-hydroxy-4-(methylthio)-butanoic acid (HMB) in the rumen [10]. The results of the present experiment indicated that supplementing HMBi at 12 or 24 g d^−1^ did not affect the rumen microbial N flow (*p* > 0.05), suggesting that HMBi did not affect the ruminal microbial N synthesis in steers.

Combined with statistical analysis and the VIP value obtained from the OPLS-DA, 32 DEMs were identified between the control group and the 24 g HMBi d^−1^ group (Table 6). The results of the plasma metabolome profiling indicated that supplementing HMBi at 24 g d^−1^ upregulated the plasma concentrations of L-Met; Met-related metabolites including betaine, Met sulfoxide, and taurine; and L-isoleucine and tyrosine (*p* < 0.05). The results demonstrated that at least part of the HMBi was absorbed and transformed into Met in the liver even though the actual amount of Met resulting from HMBi was unclear. The results are in agreement with previous studies which reported that supplementing HMBi at 0.26% DM and 0.35% DM increased the plasma concentration of Met in dairy cows [11,15]. It should be noted that supplementing HMBi at 24 g d^−1^ did not improve the N retention and the NRR in steers even though it upregulated the plasma concentration of Met. The main reasons for the insignificant effects on N utilization could be attributed to the low transport efficiency of HMBi across the rumen epithelium [32] and the steers that were entering the fattening phase. The effects of HMBi on downregulating the plasma concentrations of L-serine, glycine, diaminopimelic acid, and other metabolites (*p* < 0.05) could be another reason for the nonsignificant effects of HMBi on N utilization, even though these AA and metabolites are not essential for protein synthesis. However, the mechanisms of the effects of HMBi on these AA were unclear. 

It is usually believed that the N retention is low and the fat deposition is high in beef cattle in the fattening phase [33]. However, the N retention of the steers in the present experiment ranged between 35.1 and 40.8 g d^−1^, which was equivalent to 219.4–254.8 g CP d^−1^ and relatively high. Supplementing HMBi at 12 or 24 g d^−^^1^ did not affect the N retention and NRR of steers even though HMBi upregulated plasma relative concentration of Met. The results suggested that HMBi was inefficient to improve the N utilization in fattening beef cattle.

## 4. Conclusions

Supplementing HMBi at 12 or 24 g d^−^^1^ did not improve the N retention and NRR in steers even though HMBi upregulated the plasma relative concentration of Met. The nonsignificant effects of HMBi on improving the N retention and NRR could be mainly attributed to the fattening phase experienced by steers even though the N retention was found to be high. The results suggested that HMBi may be an inefficient Met supplement for beef cattle in the fattening period.

## Figures and Tables

**Table 1:** Ingredients and nutritional composition of the basal diet.

Items	Contents
Ingredients, % DM	
Corn silage	49.21
Corn grain	36.07
Soybean meal	6.58
Wheat bran	4.16
Corn gluten meal	3.96
Sodium bicarbonate	0.01
Sodium chloride	0.01
Nutrient composition, % DM	
OM	93.30
CP	12.53
RDP ^1^, % CP	61.85
RUP ^1^, % CP	38.15
aNDF	41.28
ADF	23.08
Met	0.15
Lys	0.43
Gross energy, MJ kg^−1^ DM	19.32
NEmf ^2^, MJ kg^−1^ DM	6.40

Abbreviations: DM, dry matter; OM, organic matter; CP, crude protein; RDP, rumen degradable protein; RUP, rumen undegradable protein; aNDF, neutral detergent fiber; ADF, acid detergent fiber; GE, gross energy; Met, methionine; Lys, lysine; NEmf, net energy for maintenance and fattening. ^1^ Calculated according to NRC [19] based on the CP fractionation of feeds determined using the methods of Licitra et al. [20]. ^2^ Calculated based on the nutritional composition of feeds according to Feng [18].

**Table 2:** Met requirement and supply of beef cattle.

Items	HMBi Supplemented, g d^−1^		
	0	12	24
Sources of metabolizable Met supply, g d^−1^			
RUP ^1^	3.20	3.20	3.20
MCP ^2^	8.15	7.50	7.97
HMBi ^3^	0	2.66	5.33
Total	11.35	13.36	16.50
Metabolizable Met requirement ^4^, g d^−1^			
Pre-trial, 593 ± 23 kg BW	11.74	11.74	11.74
Post-trial, 622 ± 20 kg BW	11.94	11.94	11.94

Abbreviations: HMBi, 2-hydroxy-4-(methylthio)-butanoic isopropyl ester; Met, methionine; MCP, microbial crude protein; RUP, rumen undegradable protein; BW, body weight. ^1^ Calculated as dry matter (DM) intake (6990 g d^−1^) × Met content in the basal diet (0.15% DM) × RUP (38.2% crude protein) × intestinal digestibility (80%) of Met according to NASEM [30]. ^2^ Calculated as microbial nitrogen synthesis × 6.25 × 0.80 × 0.80 (MCP is assumed to contain 80% true protein, which is 80% digestible) × Met content in MCP (2.6 g 100 g^−1^ protein) according to NASEM [30]. ^3^ According to HMBi product specification. ^4^ Calculated as the sum of metabolizable Met requirement for maintenance and growth (average daily gain was assumed to be 0.4 kg d^−1^) according to NASEM [30].

**Table 3:** Effects of supplementing HMBi on N metabolism in steers.

Item	HMBi Supplemented, g d^−1^			SEM	*p*-Value		
	0	12	24		Treatment	Linear	Quadratic
DMI, kg d^−1^	6.99	6.99	6.99	-	-	-	-
N intake, g d^−1^	140.19	140.19	140.19	-	-	-	-
Fecal N							
g d^−1^	51.08	53.67	53.37	1.579	0.811	0.710	0.614
% N intake	36.43	38.29	38.08	1.126	0.811	0.614	0.710
% total N excretion	51.45	52.95	50.94	1.548	0.890	0.909	0.654
Urinary N							
g d^−1^	48.35	47.60	51.68	2.059	0.747	0.570	0.633
Urinary/N intake, %	33.49	33.95	36.87	1.469	0.747	0.570	0.633
Total N excretion/N intake, %	48.54	47.04	48.21	1.548	0.890	0.909	0.654
Fecal N/urinary N	1.08	1.14	1.05	0.067	0.883	0.487	0.574
Total N excretion, g d^−1^	99.43	101.28	105.06	1.969	0.577	0.308	0.834
N retention							
g d^−1^	40.76	38.90	35.12	1.969	0.577	0.308	0.834
NRR, %	29.07	27.75	25.05	0.205	0.577	0.308	0.834

Abbreviations: HMBi, 2-hydroxy-4-methylthio-butanoic isopropyl ester; DMI, dry matter intake; N, nitrogen; SEM, standard error of mean.

**Table 4:** Effects of supplementing HMBi on urinary N compounds in steers.

Item	HMBi Supplemented, g d^−1^			SEM	*p*-Value		
	0	12	24		Treatment	Linear	Quadratic
Urea, mmol d^−1^	1072.10	1013.13	1022.38	67.412	0.472	0.415	0.370
Urea-N/urinary N	62.43	59.12	65.65	1.840	0.401	0.499	0.249
Allantoin, mmol d^−1^	121.21	111.75	116.74	9.724	0.942	0.875	0.769
Allantoin-N/urinary N	13.99	13.05	12.53	0.839	0.819	0.549	0.919
Uric acid, mmol d^−1^	6.34	8.07	8.97	0.632	0.245	0.112	0.745
Uric acid-N/urinary N	0.71	0.94	0.97	0.058	0.131	0.069	0.376
Creatinine, mmol d^−1^	105.12	98.84	117.73	5.663	0.438	0.401	0.338
Creatinine-N/urinary N	9.20	8.70	9.53	0.263	0.495	0.640	0.291
Hippuric acid, mmol d^−1^	144.73	153.46	170.44	9.868	0.622	0.357	0.860
Hippuric acid-N/urinary N	4.13	4.50	4.71	0.263	0.717	0.438	0.905
Urinary PD, mmol d^−1^	127.55	119.82	125.71	3.090	0.161	0.262	0.944
Urinary PD-N/urinary N	14.70	13.99	13.50	0.409	0.651	0.373	0.360
Rumen microbial N flow, g d^−1^	78.36	72.07	76.59	2.416	0.144	0.354	0.787

Abbreviations: HMBi, 2-hydroxy-4-methylthio-butanoic isopropyl ester; N, nitrogen; PD, purine derivatives; SEM, standard error of mean.

**Table 5:** Effects of supplementing HMBi on plasma biochemical indices in steers.

Item	HMBi Supplemented, g d^−1^			SEM	*p*-Value		
	0	12	24		Treatment	Linear	Quadratic
Total protein, g L^−1^	72.97	73.25	73.73	1.255	0.864	0.629	0.847
Albumin, g L^−1^	28.50	26.26	28.21	0.851	0.572	0.896	0.314
Glucose, mmol L^−1^	4.18	4.29	4.69	0.125	0.241	0.120	0.567
Total cholesterol, mmol L^−1^	2.73	2.26	2.06	0.177	0.317	0.154	0.731
Triglyceride, mmol L^−1^	1.19	1.21	1.19	0.018	0.937	0.946	0.735
Urea, mmol L^−1^	4.01	3.79	4.14	0.228	0.857	0.844	0.620
Uric acid, μmol L^−1^	33.96	33.05	31.97	0.816	0.672	0.393	0.965
Creatinine, μmol L^−1^	152.61	143.11	125.31	6.814	0.281	0.131	0.769

Abbreviations: HMBi, 2-hydroxy-4-methylthio-butanoic isopropyl ester; SEM, standard error of mean.

**Table 6:** Differential plasma metabolites in steers supplemented with 0 and 24 g HMBi d^−1^ using a VIP threshold of 1.

Super Class	Metabolites	VIP	FDR
Upregulated			
Organic acids and derivatives	Betaine	1.26	<0.05
Organic acids and derivatives	L-methionine	1.75	<0.05
Organic acids and derivatives	L-isoleucine	1.42	<0.05
Organic acids and derivatives	Methionine sulfoxide	1.38	<0.05
Organic acids and derivatives	Taurine	1.71	<0.05
Organic acids and derivatives	Tyrosine	1.12	<0.05
Lipids and lipidlike molecules	Phytanic acid	1.44	<0.05
Lipids and lipidlike molecules	Tocopherol	1.68	<0.05
Lipids and lipidlike molecules	Epiandrosterone	1.37	<0.05
Lipids and lipidlike molecules	Retinol	1.63	<0.05
Lipids and lipidlike molecules	LysoPC(18:1(9Z))	1.55	<0.05
Organoheterocyclic compounds	Xanthine	1.27	<0.05
Organoheterocyclic compounds	Hypoxanthine	1.12	<0.05
Organoheterocyclic compounds	Guanine	1.28	<0.05
Organoheterocyclic compounds	Guanine	1.46	<0.05
Organoheterocyclic compounds	Riboflavin	1.52	<0.05
Organic nitrogen compounds	Sphingosine	1.56	<0.05
Organic nitrogen compounds	Thiamine pyrophosphate	1.62	<0.05
Phenylpropanoids and polyketides	Cinnamic acids	1.76	<0.05
Downregulated			
Organic acids and derivatives	L-serine	1.56	<0.05
Organic acids and derivatives	Glycine	1.42	<0.05
Organic acids and derivatives	Diaminopimelic acid	1.50	<0.05
Organic acids and derivatives	3-Methylhistidine	1.62	<0.05
Organic acids and derivatives	L-pipecolic acid	1.70	<0.05
Lipids and lipidlike molecules	LysoPC(18:3(9Z,12Z,15Z))	1.41	<0.05
Lipids and lipidlike molecules	Fucoxanthin	1.25	<0.05
Benzenoids	Phenylacetic acid	1.63	<0.05
Organic nitrogen compounds	Putrescine	1.48	<0.05
Organic nitrogen compounds	Acetylcholine	1.39	<0.05
Organoheterocyclic compounds	4,5-Dimethyloxazole	1.28	<0.05
Organoheterocyclic compounds	Serotonin	1.46	<0.05
Organic oxygen compounds	Validamycin B	1.62	<0.05
Organic oxygen compounds	Gluconic lactone	1.37	<0.05
Phenylpropanoids and polyketides	Naringenin	1.19	<0.05

Abbreviations: HMBi, 2-hydroxy-4-methylthio-butanoic isopropyl ester; VIP, variable importance in the projection; FDR, false discovery rate.

## Data Availability

Not applicable.
